# Admixture Mapping of 15,280 African Americans Identifies Obesity Susceptibility Loci on Chromosomes 5 and X

**DOI:** 10.1371/journal.pgen.1000490

**Published:** 2009-05-22

**Authors:** Ching-Yu Cheng, W. H. Linda Kao, Nick Patterson, Arti Tandon, Christopher A. Haiman, Tamara B. Harris, Chao Xing, Esther M. John, Christine B. Ambrosone, Frederick L. Brancati, Josef Coresh, Michael F. Press, Rulan S. Parekh, Michael J. Klag, Lucy A. Meoni, Wen-Chi Hsueh, Laura Fejerman, Ludmila Pawlikowska, Matthew L. Freedman, Lina H. Jandorf, Elisa V. Bandera, Gregory L. Ciupak, Michael A. Nalls, Ermeg L. Akylbekova, Eric S. Orwoll, Tennille S. Leak, Iva Miljkovic, Rongling Li, Giske Ursin, Leslie Bernstein, Kristin Ardlie, Herman A. Taylor, Eric Boerwinckle, Joseph M. Zmuda, Brian E. Henderson, James G. Wilson, David Reich

**Affiliations:** 1 Department of Epidemiology, Johns Hopkins University, Baltimore, Maryland, United States of America; 2 Department of Ophthalmology, National Yang Ming University School of Medicine, Taipei, Taiwan; 3 Taipei Veterans General Hospital, Taipei, Taiwan; 4 Welch Center for Prevention, Epidemiology and Clinical Research, Johns Hopkins University, Baltimore, Maryland, United States of America; 5 Program in Medical and Population Genetics, Broad Institute of Harvard and MIT, Cambridge, Massachusetts, United States of America; 6 Department of Genetics, Harvard Medical School, Boston, Massachusetts, United States of America; 7 Department of Preventive Medicine, Keck School of Medicine, University of Southern California, Los Angeles, California, United States of America; 8 Laboratory of Epidemiology, Demography and Biometry, National Institute on Aging, Bethesda, Maryland, United States of America; 9 Department of Clinical Sciences, University of Texas Southwestern Medical Center, Dallas, Texas, United States of America; 10 McDermott Center for Human Growth and Development, University of Texas Southwestern Medical Center, Dallas, Texas, United States of America; 11 Donald W. Reynolds Cardiovascular Clinical Research Center, University of Texas Southwestern Medical Center, Dallas, Texas, United States of America; 12 Northern California Cancer Center, Fremont, California, United States of America; 13 Department of Health Research and Policy, Stanford University School of Medicine, Stanford, California, United States of America; 14 Stanford Cancer Center, Stanford, California, United States of America; 15 Department of Cancer Prevention and Control, Roswell Park Cancer Institute, Buffalo, New York, United States of America; 16 Department of Pathology, Keck School of Medicine, University of Southern California, Los Angeles, California, United States of America; 17 Department of Pediatrics, Johns Hopkins University, Baltimore, Maryland, United States of America; 18 Department of Medicine, Johns Hopkins University, Baltimore, Maryland, United States of America; 19 Department of Health Policy and Management, Johns Hopkins University, Baltimore, Maryland, United States of America; 20 Department of Biostatistics, Johns Hopkins University, Baltimore, Maryland, United States of America; 21 Department of Medicine, University of California San Francisco, San Francisco, California, United States of America; 22 Institute for Human Genetics, University of California San Francisco, San Francisco, California, United States of America; 23 Helen Diller Family Comprehensive Cancer Center, University of California San Francisco, San Francisco, California, United States of America; 24 Department of Anesthesia and Perioperative Care, University of California San Francisco, San Francisco, California, United States of America; 25 Department of Medical Oncology, Dana–Farber Cancer Institute, Boston, Massachusetts, United States of America; 26 Department of Oncological Sciences, Mount Sinai School of Medicine, New York, New York, United States of America; 27 The Cancer Institute of New Jersey, Robert Wood Johnson Medical School, New Brunswick, New Jersey, United States of America; 28 Molecular Genetics Section, Laboratory of Neurogenetics, Intramural Research Program, National Institute on Aging, Bethesda, Maryland, United States of America; 29 Jackson Heart Study Analysis Group, Jackson State University, Jackson, Mississippi, United States of America; 30 Oregon Clinical and Translational Research Institute, Oregon Health and Science University, Portland, Oregon, United States of America; 31 Department of Epidemiology, Graduate School of Public Health, University of Pittsburgh, Pittsburgh, Pennsylvania, United States of America; 32 Department of Preventive Medicine, Division of Biostatistics and Epidemiology, University of Tennessee, Memphis, Tennessee, United States of America; 33 Department of Nutrition, University of Oslo, Oslo, Norway; 34 Department of Cancer Etiology, Division of Population Science, City of Hope National Medical Center, Duarte, California, United States of America; 35 Genomics Collaborative, Cambridge, Massachusetts, United States of America; 36 Jackson State University, Jackson, Mississippi, United States of America; 37 Tougaloo College, Tougaloo, Mississippi, United States of America; 38 University of Mississippi Medical Center, Jackson, Mississippi, United States of America; 39 Human Genetics Center, University of Texas Health Science Center at Houston, Houston, Texas, United States of America; 40 G. V. (Sonny) Montgomery Veterans Affairs Medical Center, Jackson, Mississippi, United States of America; University of Oxford, United Kingdom

## Abstract

The prevalence of obesity (body mass index (BMI) ≥30 kg/m^2^) is higher in African Americans than in European Americans, even after adjustment for socioeconomic factors, suggesting that genetic factors may explain some of the difference. To identify genetic loci influencing BMI, we carried out a pooled analysis of genome-wide admixture mapping scans in 15,280 African Americans from 14 epidemiologic studies. Samples were genotyped at a median of 1,411 ancestry-informative markers. After adjusting for age, sex, and study, BMI was analyzed both as a dichotomized (top 20% versus bottom 20%) and a continuous trait. We found that a higher percentage of European ancestry was significantly correlated with lower BMI (ρ = −0.042, *P* = 1.6×10^−7^). In the dichotomized analysis, we detected two loci on chromosome X as associated with increased African ancestry: the first at Xq25 (locus-specific LOD = 5.94; genome-wide score = 3.22; case-control Z = −3.94); and the second at Xq13.1 (locus-specific LOD = 2.22; case-control Z = −4.62). Quantitative analysis identified a third locus at 5q13.3 where higher BMI was highly significantly associated with greater European ancestry (locus-specific LOD = 6.27; genome-wide score = 3.46). Further mapping studies with dense sets of markers will be necessary to identify the alleles in these regions of chromosomes X and 5 that may be associated with variation in BMI.

## Author Summary

Obesity is about 1.5-fold more prevalent in African Americans than European Americans. To determine whether genetic background may contribute to this observed disparity, we scanned the genomes of African Americans, searching for genomic regions where obese individuals have a difference from the average proportion of African ancestry. By examining genetic data from more than 15,000 African Americans, we show that the proportion of European ancestry is inversely correlated with BMI. In obese individuals, we detect two loci with increased African ancestry on chromosome X (Xq13.1 and Xq25) and one locus with increased European ancestry on chromosome 5 (5q13.3). The 5q13.3 and Xq25 regions both contain genes that are known to be involved in appetite regulation. Our results suggest that genetic factors may contribute to the difference in obesity prevalence between African Americans and European Americans. Further studies of the regions may identify the causative variants affecting susceptibility to obesity.

## Introduction

Obesity is a highly prevalent condition that increases the risk of many illnesses such as cardiovascular disease, diabetes, and some cancers. Familial aggregation studies have shown that both genetic and environmental factors are involved in the development of common forms of obesity, and heritability estimates suggest that approximately 40% of variation in body mass index (BMI) can be attributed to genetic factors [1],[2].

The current increase in prevalence of obesity in the U.S. has been hypothesized to be the result of genetic susceptibility in an environment that promotes obesity [3]. James V. Neel in 1962 proposed the “thrifty gene hypothesis” to put these epidemiological observations in an evolutionary context [4]. He suggested that the genetic factors that predispose to weight gain might have been selectively advantageous in ancient environments where food was scarce, but might have become deleterious in modern environments where food is plentiful and lifestyles are generally sedentary. Based on epidemiologic evidence, specific racial/ethnic groups seem to be particularly susceptible to obesity in the U.S., especially African Americans, Pima Indians, and Pacific Islanders [3],[5]. Data from the 2003–2004 National Health and Nutritional Examination Survey (NHANES) indicate that African Americans are about 1.5 times more likely to be obese (defined as BMI ≥30 kg/m^2^) than European Americans even in homogeneous socioeconomic groups [6],[7].

Recent genome-wide association studies have shown that variants in the fat mass and obesity-related gene (*FTO*) are significantly associated with obesity in populations of European origin [8]–[10]. It was estimated that a ∼0.4 kg/m^2^ rise in BMI is associated with each copy of the A allele at rs9939609 in populations of European descent [8]. While the association was replicated in East Asian populations [11]–[13], no association was observed in African Americans [9], although there is evidence that another SNP (rs3751812) affects the risk of obesity in African Americans as well [14]. These results suggest that the genetic factors predisposing to obesity in African Americans at *FTO* may be different from that in other populations, although an alternative explanation for these observations is that the causal variant has not been identified, and that the linkage disequilibrium patterns to the causal variant are different in African and non-African populations.

To screen for genetic variants modulating BMI in African Americans, we used admixture mapping, a technique that scans the genomes of recently admixed populations and searches for genomic regions in people with disease where there is substantial deviation in one of the parental ancestries compared with the genome average [15]–[20]. To maximize power to detect variants affecting BMI, we carried out a pooled admixture mapping analysis of 15,280 African-American samples from 14 studies, including the Atherosclerosis Risk in Communities (ARIC) Study, the Breast Cancer Family Registry (BCFR), the Los Angeles component of the Women's Contraceptive and Reproductive Experiences (CARE) Study, the Dallas Heart Study (DHS), the Family Investigation of Nephropathy and Diabetes (FIND) Study, the Genomics Collaborative (GCI) Study, the Health, Aging and Body Composition (Health ABC) Study, the Jackson Heart Study (JHS), the Learning the Influence of Family and the Environment (LIFE) Study, the Multiethnic Cohort of Los Angeles and Hawaii (MEC), the Osteoporotic Fractures in Men Study (MrOS), the San Francisco Bay Area Breast Cancer Study (SFBABCS), the Study of Osteoporotic Fractures (SOF), and the Women's Circle of Health Study (WCHS).

## Methods

### Study Populations and SNP Genotyping

Our analysis was carried out in 15,280 African Americans. Samples were scanned with at least one of three iteratively improved and partially overlapping panels of ancestry-informative markers. The Phase 1 panel was published in Smith et al. 2004 [20] and Reich et al. 2005 [21]. The Phase 2 panel was first published in Reich et al. 2007 [22]. The Phase 3 panel was first published in Nalls et al. 2008 [23] (http://www.illumina.com/downloads/AfricanAmericanAdmixture_DataSheet.pdf). Altogether 4,372 markers were genotyped in the present study, with a median of 1,411 markers genotyped per sample. We found in practice that all marker panels provided at least 60% of the maximum possible information about ancestry.

The samples were assembled from 14 studies (Table 1). Of these, six (ARIC, DHS, Health ABC, JHS, MrOS and SOF) were prospective cohort studies that did not oversample any particular phenotype, and eight (BCFR, CARE, FIND, GCI, LIFE, MEC, SFBABCS and WCHS) were studies that oversampled individuals with particular phenotypes, such as breast cancer, end-stage renal disease, type 2 diabetes, hypertension, and prostate cancer. Brief description of each study as well as the number of samples we analyzed after applying various data quality filters are provided in Text S1.

**Table 1:** Characteristics of 15,280 African American adults by study population.

									Diabetes status	
Study	No. of samples	DNA source	Phase of marker panel	Female, %	Age (yrs)	European ancestry from autosomes, %	European ancestry from the X chromosome, %	BMI (Kg/m^2^)	No. with information on diabetes status	Diabetes, %
ARIC	3,522	genomic	3	62.1	53.5±5.8	17.6±10.2	14.2±7.4	29.6±6.2^a^	3,450	19.5
BCFR^b^	268	genomic	2	100.0	50.3±9.4	22.7±12.4	17.9±9.2	30.3±6.7^a^	0	-
CARE^b^	365	WGA	3	100.0	48.9±8.0	22.0±11.5	17.3±8.4	27.7±6.1	0	-
DHS	1,718	genomic	1	57.5	44.8±10.2	16.2±8.2	13.1±6.1	31.5±8.2^a^	1,718	13.6
FIND^b^	1,445	genomic	3	50.4	48.4±12.4	17.1±8.4	13.9±6.1	28.8±7.2	1,445	22.0
GCI^b^	503	genomic	2	54.5	57.9±13.6	15.3±11.2	12.6±8.2	31.6±7.0	503	8.9
Health ABC	1,172	WGA	2	57.1	73.4±2.9	20.9±12.8	16.7±9.4	28.5±5.2^a^	1,164	21.4
JHS	2,141	genomic	2/3	60.0	52.4±11.1	17.9±9.2	14.5±6.7	31.9±7.1^a^	2,106	17.9
LIFE^b^	108	WGA	3	100.0	42.2±5.3	21.3±11.0	17.1±7.9	29.0±6.9	0	-
MEC^b^	3,199	genomic	1/2/3	31.3	62.7±8.1	23.4±14.0	18.4±10.1	28.3±5.3	1,551	58.9
MrOS	199	WGA	3	0.0	71.7±5.2	21.2±13.6	16.9±9.9	28.5±4.4^a^	182	27.5
SFBABCS^b^	152	genomic	2	100.0	55.1±11.8	22.6±14.7	17.9±11.0	30.5±6.0^a^	0	-
SOF	368	WGA	3	100.0	75.0±4.7	24.4±13.8	19.1±10.1	29.9±5.8^a^	368	16.3
WCHS^b^	120	genomic	2	100.0	50.1±9.3	16.7±14.8	13.6±11.2	30.3±6.5^a^	0	-
Total	15,280	-	-	55.7	56.0±12.2	19.3±11.5	15.5±8.4	29.8±6.6	12,487	23.4

Ranges are given in terms of ±1 standard deviation. ARIC, Atherosclerosis Risk in Communities Study; BCFR, Breast Cancer Family Registry; CARE, Los Angeles component of the Women's Contraceptive and Reproductive Experiences Study, DHS, Dallas Heart Study; FIND, Family Investigation of Nephropathy and Diabetes Study; GCI, Genomics Collaborative Study; Health ABC, Health, Aging and Body Composition Study; JHS, Jackson Heart Study; LIFE, Learning the Influence of Family and the Environment Study; MEC, Multiethnic Cohort of Los Angeles and Hawaii; MrOS, Osteoporotic Fractures in Men Study; SFBABCS, the San Francisco Bay Area Breast Cancer Study; SOF, Study of Osteoporotic Fractures; WCHS, Women's Circle of Health Study; WGA, whole genome amplification.

a BMI were measured in an actual clinical visit in the six prospective cohort studies and in the BCFR, SFBABCS and WCHS; for others, BMI was calculated from self-reported weight and height.

b These studies include case-control studies and so are not a representative cross-section of the populations. BCFR, CARE, LIFE, SFBABCS and WCHS oversampled women with breast cancer. FIND oversampled individuals with nephropathy. GCI focused on individuals with hypertension. MEC oversampled individuals with type 2 diabetes, prostate cancer, breast cancer, and hypertension.

In the six prospective cohort studies, anthropometric measurements were performed using study-specific standardized protocols, and BMI was calculated as weight (in kg) divided by height (in meters) squared. In the BCFR, SFBABCS and WCHS, BMI was also calculated from height and weight measures taken at the time of study interview by trained research staff. In the remaining studies, BMI was calculated using self-reported weight and height.

### Estimates of Allele Frequencies in West African and European American Ancestral Populations

We used previously published genotyping data to estimate the frequency of each SNP in West Africans and European Americans [20],[24],[25]. We only used SNPs for which we were able to obtain data from both West African (Yoruba) and European American (CEU) populations from the International Haplotype Map. For SNPs in the Phase 1 panel, we also added additional genotyping data from African and European samples, which was the same as the data collected in Smith et al. 2004 [20].

### SNP Filters

To decrease the likelihood of false-positives in our admixture scans, we applied a series of filters that had the goal of detecting and removing any SNPs with problematic genotyping, as described previously [20]–[22]. Briefly, we applied three previously published filters. (1) We applied a “mapcheck” filter that tests whether the estimate of ancestry obtained based on the information from that SNP alone is consistent with the estimate of ancestry obtained from neighboring markers; SNPs with discrepancies are removed from analysis. (2) We applied a “freqcheck” filter that tests whether the observed frequency of a SNP in African Americans is statistically consistent with being a mixture of the frequencies observed in the West Africans and European American samples that we used to represent the ancestral populations. (3) We finally applied an “ldcheck” filter that for each sample, iteratively removes SNPs that are less informative (in terms of the information content about ancestry) until none are within 200 kilobases of each other or are in detectable linkage disequilibrium with each other in the ancestral West African or European American populations [21],[25].

### Elimination of Samples with Incomplete Genotype and Phenotype Data

We required all individuals included in the study to have complete phenotypic information, including BMI, age at the time of measurement, and gender. We also required all individuals to have a full admixture scan, and we removed samples that were outliers with respect to others in the same cohort in the sense of having many fewer genotypes, as we found that this predicts less reliable data. The data for the great majority of the samples we analyze in this study was reported previously [22]–[26], and hence we do not report further details of the sample genotyping here.

### Estimating Local and Genome-Wide Ancestry in the African American Samples

We estimated the European and African ancestry at each locus and genome-wide using the ANCESTRYMAP software [19]. ANCESTRYMAP uses a Hidden Markov Model (HMM) to combine the weak information about local ancestry that is provided by each marker, into a more confident estimate that takes into account the information from many neighboring markers. The HMM is nested within a Markov Chain Monte Carlo method, which accounts for uncertainty in the unknown parameters: SNP allele frequencies in the West African and European American ancestral populations, the number of generations since mixture and the average proportion of ancestry inherited from ancestral populations. All Markov Chain Monte Carlo runs used 100 burn-in and 200 follow-on iterations, as previously recommended [19], except for one longer run of 1,000 burn-in and 2,000 follow-on iterations, which we carried out to check the stability of our results. Samples with an estimated percentage of European ancestry of more than 0.85 (n = 27) were excluded from this analysis.

### Calculation of Covariate-Adjusted BMI

Body mass index was defined as described above. For most of our admixture analysis runs, BMI was adjusted for age, age-squared, sex and study, using multivariate linear regression analyses, and the residuals that emerged from this regression analysis were used for subsequent analysis.

### Admixture Mapping Scans Treating BMI as a Dichotomous Trait

ANCESTRYMAP [19] was used to test whether individuals with high or low BMI had a proportion of ancestry that was significantly different from the genome average in the same samples.

For the dichotomous admixture scans, we defined the top 20% of samples with the highest residuals of BMI as cases and the bottom 20% as controls. Because a prior distribution on risk models is required for the Bayesian statistical analysis in ANCESTRYMAP [19], we tested a total of 24 pre-specified risk models and assessed overall evidence of association by averaging all models. The first eight models specified 0.5-, 0.6-, 0.7-, 0.8-, 1.3-, 1.5-, 1.7- and 2.0-fold increased risk due to inheritance of one copy of European ancestral allele for cases, with a control risk of 1. The next eight models used the same set of risk models for cases, and the control risks were set to be the reciprocal of the case risks. The last eight models used a case risk of 1, but specified that controls had risks of 0.5, 0.6, 0.7, 0.8, 1.3, 1.5, 1.7 and 2.0. These risk models equally tested for both positive and negative associations of BMI with African ancestry [19].

To assess statistical significance, the ANCESTRYMAP software provided two scores: a *locus-specific score* and a *case-control score*. A *locus-specific score* is obtained in cases (i.e., case-only analysis) by calculating the likelihood of the genotyping data at the SNPs at the locus under the risk model and comparing it to the likelihood of the genotyping data at the SNPs at the locus assuming that the locus is uncorrelated to the phenotype [19]. The ratio of these two likelihoods is the “likelihood ratio”, and the log-base-10 of this quantity is the “LOD” score. A locus-specific LOD score of >5 has been recommended as criterion for genome-wide significance and >4 has been recommended as a criterion for genome-wide suggestiveness [27].

To obtain an assessment of the evidence for a risk locus anywhere in the genome—which we call the “genome-wide score”—we averaged the likelihood ratio for association across all loci in the genome, and took the log10 to obtain a genome-wide score. We interpret a genome-wide score>2 as significant and >1 as suggestive as previously recommended [27].

A *case-control score* was calculated by comparing locus-specific deviations in European ancestry in cases versus controls at each locus across the genome. This score tests whether any deviation in ancestry from the genome-wide average is significantly different comparing cases with controls [19]. If there is no locus associated with disease, the case-control score is expected to be distributed approximately according to a standard normal distribution. For loci identified by this score, the level of genome-wide significance was defined as a case-control Z score<−4.06 or >4.06 (i.e., an uncorrected nominal *P*<5×10^−5^, or a corrected nominal *P*<0.05 after conservatively correcting for 1,000 hypotheses tested, corresponding to independent chromosomal chunks assigned to either African or European ancestry). The case-control score is particularly important for X chromosome analyses. Case-only admixture analyses of the X chromosome are complicated by the fact that African Americans tend to have lower proportions of European ancestry on the X chromosome than on the autosomes, and thus an X-chromosome-wide-specific estimate of ancestry is required [19]. However, such an X-chromosome-wide estimate of ancestry is difficult to obtain because of the relatively short size of the X chromosome. By contrast, a case-control score is robust to uncertainty in the X-chromosome-wide European ancestry proportion. A systematic bias in the estimate of ancestry at a locus is expected to affect controls as much as cases, and hence is not expected to generate a significant difference between cases and controls.

### Quantitative Admixture Mapping Scans

We have now extended ANCESTRYMAP to also allow for association analyses of quantitative traits (Text S2). Briefly, we applied a normal-quantile transformation to the covariate-adjusted BMI to obtain normally distributed values for subsequent quantitative admixture scans and regression-based association analysis. To test for association to a quantitative trait, we applied a feature, “qtmode”, in ANCESTRYMAP (see Text S2 for mathematical details). In qtmode, each risk model represented a correlation coefficient (ρ) of European ancestry with the normally distributed value of the trait. For this analysis, we tested equally spaced risk models of ρ = 0.1, 0.08, 0.06, 0.04, 0.02, −0.02, −0.04, −0.06, −0.08 and −0.1. To determine statistical significance, we used the same thresholds of locus-specific LOD and genome-wide scores as described above for the dichotomous analyses.

### Credible Interval for the Position of a Genetic Locus

To calculate a 95% credible interval (CI) for the position of a locus, we obtained the likelihood ratio for association at each marker across the chromosome where we found an association. This provided a Bayesian posterior probability for the position of the underlying causal variant assuming a flat prior distribution across the region for the position of the disease locus. The central region of this peak containing 95% of the area was used as the CI.

### Assessing Associations of BMI to Local Ancestry at the Admixture Peak

Local estimates of ancestry at the admixture peak were obtained using the ANCESTRYMAP software [19]. Heterogeneity of the correlations between the local ancestry and BMI across studies was quantified using the *I* ^2^ inconsistency metric [28]. To determine the association of BMI with local ancestry at the admixture peak, we performed a linear regression analysis, with the transformed BMI as the dependent variable and the local estimates of ancestry as independent variables. To determine whether there was evidence of residual association with local ancestry after adjustment for global ancestry, we included each individual's percentage of genome-wide European ancestry as a covariate in the regression models. This enabled us to increase power by including all samples in a quantitative analysis, rather than using only a subset of samples with the highest 20% and lowest 20% values in the dichotomous admixture scans described above.

### Ethics Statement

This study was conducted according to the principles expressed in the Declaration of Helsinki. All sample collections were carried out according to institutionally approved protocols for study of human subjects and written informed consent was obtained from all subjects.

## Results

The demographic and phenotypic characteristics of the 15,280 African Americans included in the admixture scan are summarized in Table 1. Because the individual studies differed in aims, design and methods of data collection, there was considerable variation across studies in the distribution of age, sex, BMI, and frequency of diabetes. For example, there was an extremely high proportion of type 2 diabetes among the MEC samples (58.9%), reflecting the fact that these samples included a group of cases with type 2 diabetes who had been specifically genotyped as part of an admixture scan (http://www.broad.mit.edu/node/549). Combined across all studies, the mean BMI was 29.8±6.6 kg/m^2^, and 40.2% of the population had BMI ≥30 kg/m^2^. Mean BMI differed significantly across studies (*P*<0.001).

### Estimates of European Ancestry

The average percentage of genome-wide European ancestry in these samples was 19.3±11.5% based on estimates from the autosomes, and the average percentage of European ancestry on the X chromosome was 15.5±8.4%. Because the study samples came from different resources and locations across the U.S., there was significant variation in average European ancestry, either estimated from autosomes or the X chromosome, across studies (*P*<0.001).

### Percent European Ancestry Was Inversely Associated with BMI among African Americans

**Figure 1:**
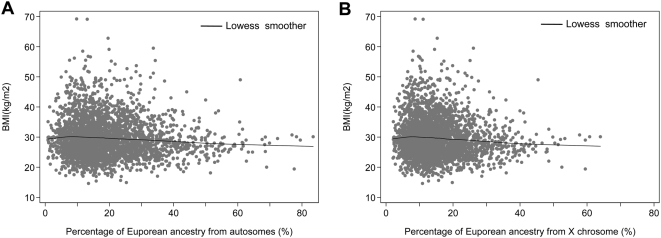
Scatter-plots of BMI vs. the estimated percentage of European ancestry. (A) Percentage of European ancestry was estimated based on the autosomes. (B) Percentage of European ancestry was estimated based on chromosome X. Data are plotted using 20% of the samples (selected at random) for better visualization.

The relationship between BMI and percentage of European ancestry is shown in Figure 1. BMI was inversely correlated with European ancestry as estimated from autosomes, an effect that was weak (ρ = −0.042) but statistically significant (*P* = 1.6×10^−7^) given the large sample size. It was also significantly correlated with European ancestry as estimated from the X chromosome (ρ = −0.046, *P* = 1.2×10^−8^).

### Dichotomous Admixture Scans Identified Two Signals on Chromosome X

**Figure 2:**
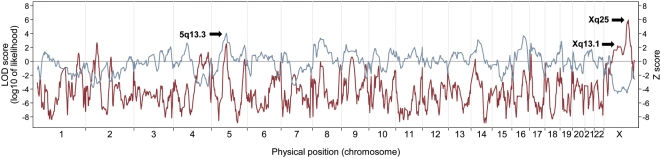
The dichotomous admixture scans for genetic loci affecting BMI. The locus-specific LOD score (red line) and the case-control Z score (blue gray line) are shown for Run 1 in Table 2: BMI was adjusted for age, age-squared, sex and studies. A signal at genome-wide significant level (locus-specific LOD = 5.94) was detected at Xq25. The Xq25 peak was also supported by the case-control statistic (Z score = −3.94, *P* = 8.1×10^−5^). Another peak on chromosome X was observed at Xq13.1 (locus-specific LOD = 2.22). Although its LOD score did not reach statistical significance, it had the largest magnitude case-control Z score of −4.62 (*P* = 3.8×10^−6^) anywhere in the genome. Moreover, we observed an admixture signal at 5q13.3 (locus-specific LOD = 2.48), which did not reach significance, but had the strongest positive case-control Z score across the genome (Z score = 4.03, *P* = 5.6×10^−5^).

The dichotomous admixture scans detected evidence of genome-wide significant associations between markers on the X chromosome and higher BMI (Table 2 and Figure 2). By comparing the 20% of samples with the highest and lowest covariate-adjusted BMI, we identified the strongest association for high BMI at Xq25 (locus-specific LOD = 5.94). The genome-wide score was 3.22, substantially exceeding our genome-wide threshold for significance. At the same locus, we also observed a case-control Z score of −3.94 standard deviations (nominal *P* = 8.1×10^−5^), which also supported an association at this locus, with obese cases having lower European ancestry than non-obese controls. Interestingly, we found another admixture peak at Xq13.1. Although the LOD score at Xq13.1 was far from significant (locus-specific LOD = 2.22), this locus had the strongest case-control Z score, −4.62 (nominal *P* = 3.8×10^−6^), anywhere in the genome. The associations at Xq13.1 was nominally genome-wide significant (*P* = 3.8×10^−3^) after conservatively correcting for 1,000 hypotheses tested.

**Table 2:** Summary of results from dichotomous admixture scans for BMI.

					Xq25		Xq13.1		5q13.3	
Run	Description	No. of cases/controls^a^	No. of SNPs^b^	Genome-wide score	Peak LOD score	Case-control Z score	Peak LOD score	Case-control Z score	Peak LOD score	Case-control Z score
1	All African Americans	3,055/3,056	3,902	3.22^c^	5.94^d^	−3.94	2.22	−4.62	2.48	4.03
2	10×more iterations for run 1	3,055/3,056	3,902	3.10^c^	5.80^d^	−3.94	2.07	−4.62	2.12	4.02
3	African Americans with information on diabetes status, BMI additionally adjusted for diabetes	2,496/2,498	3,757	3.98^c^	6.92^d^	−3.25	2.55	−4.57	2.58	4.23
4	African Americans in the six prospective cohort studies^e^	1,824/1,825	3,543	3.03^c^	6.00^d^	−3.78	1.68	−4.19	2.24	3.02
5	Non-diabetic African Americans	1,914/1,914	3,737	1.46	4.21	−3.13	2.25	−4.28	1.50	3.05
6	Male African Americans	1,351/1,353	3,716	1.38	0.96	−2.69	4.40	−4.12	0.40	1.60
7	Female African Americans	1,703/1,705	3,646	1.36	4.15	−3.00	−1.06	−2.10	−0.23	3.30
8	All African Americans, drop every even SNP	3,055/3,056	2,057	2.02^c^	4.78	−4.30	1.29	−4.74	1.58	3.80
9	All African Americans, drop every odd SNP	3,055/3,056	2,049	1.29	4.10	−3.83	0.90	−4.78	1.34	3.24
10	Best-fit multiplicative model for run 1 (0.73 multiplicative risk for chromosome X)	3,055/3,056	3,902	4.57^c^	7.24^d^	−3.94	2.71	−4.62	−	4.03
11	Best-fit multiplicative model for run 3 (0.70 multiplicative risk for chromosome X)	2,496/2,498	3,757	5.25^c^	8.16^d^	−3.25	3.25	−4.57	−	4.23

BMI was adjusted for age, age-squared, sex and study for all runs, except for runs 6 and 7, where analysis was performed in each gender group and thus not adjusted for sex.

a Cases: 20% with the highest covariate-adjusted BMI; controls: 20% with the lowest values.

b The number of SNPs analyzed after applying a series of quality filters.

c Genome-wide scores>2 are formally significant; scores>1 are suggestive.

d LOD scores>5 are formally significant; scores>4 are suggestive.

e The six cohorts composed 94% of all samples with clinically measured BMI.

To examine the potential impact of heterogeneity across the studies on our admixture-generated signals, we carried out a series of subgroup analyses (Table 2). When BMI was adjusted for diabetes in samples with information on diabetes status, the association signal at Xq25 grew stronger, with the locus-specific LOD score rising to 6.92, the genome-wide score rising to 3.98, and the case-control Z-score becoming less significant at −3.25. To take into account potential measurement errors from self-reported BMI in 40% of the samples, we also performed admixture scans restricting the samples to those from the six prospective cohort studies where BMI was clinically measured. Similarly strong evidence of association at Xq25 (locus-specific LOD = 6.00; genome-wide score = 3.03) was found. We also carried out an analysis in which we excluded individuals with diabetes to avoid problems related to co-morbidity and treatment. After removing these samples (a drop of 23.4% of the sample size), the signal of association became weaker but remained suggestive (locus-specific LOD = 4.21).

Because the admixture peaks we identified were located on chromosome X, which has a different copy number in men and women, we also performed analyses for each gender separately to explore whether the strength of association differed significantly between males and females. We found that the evidence of association at Xq25 was stronger in females (locus-specific LOD = 4.15; N = 1,703) than in males (locus-specific LOD = 0.96; N = 1,351), and that the association signal at Xq13.1 in males grew stronger with the local LOD score rising to 4.40 (Run 6 and 7 in Table 2). In the more comprehensive linear regression analysis of local ancestry, there was a significant gender difference at Xq13.1 (*P*<0.026; see below for details).

In addition to the two peaks on chromosome X, using dichotomous admixture scans we observed a few interesting regions (Figure 2 and Table S1), particularly locus 5q13.3 (locus-specific LOD = 2.48, Table 2). This locus is unique in that even though its LOD score was far from statistical significance, it had the strongest increase in European ancestry in individuals with high BMI compared to individuals with low BMI (case-control Z score = 4.03, nominal *P* = 5.6×10^−5^). The case-control score was marginally significant at genome-wide level, suggesting that higher BMI was, though counter-intuitively, associated with greater European ancestry at 5q13.3 locus.

### Quantitative Admixture Scans Detected a Third Locus on Chromosome 5

**Figure 3:**
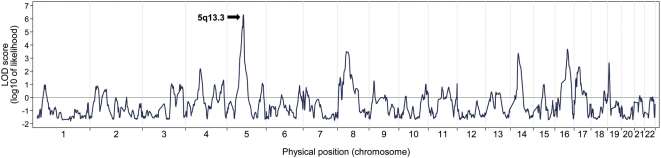
The quantitative admixture scans for genetic loci affecting BMI. The quantitative admixture scans identified an association peak at 5q13.3 with a locus-specific LOD score of 6.27 and a genome-wide score of 3.46, both reaching the thresholds for genome-wide significance.

By including all African-American samples and using BMI as a continuous trait, our quantitative admixture scan supported and strengthened the evidence of association at 5q13.3 locus (Figure 3). The peak locus-specific LOD score was 6.27 and the genome-wide score was 3.46, both reaching the thresholds for genome-wide significance.

### Evidence of Association between Admixture-Generated Signals and Continuous BMI

The local estimate of European ancestry was also extracted for each individual at each of the three admixture peaks and analyzed for association with continuous BMI (Model 1 in Table 3). Higher local European ancestry both at Xq13.1 and Xq25 was significantly and inversely associated with lower values of transformed BMI (*P* = 2.2×10^−11^ and *P* = 4.5×10^−10^, respectively). To examine whether these associations could be fully explained by the significant association between BMI and genome-wide ancestry (discussed above), we further adjusted for genome-wide European ancestry in the multivariate analysis. The residual association of local ancestry with BMI after adjusting for genome-wide ancestry remained significant at both Xq13.1 (*P* = 1.9×10^−7^) and Xq25 (*P* = 4.1×10^−6^) (Model 2 in Table 3), indicating that local ancestry had an effect on BMI above and beyond genome-wide ancestry. Both associations were nominally genome-wide significant (*P* = 1.9×10^−4^ and *P* = 4.1×10^−3^) after conservatively correcting for 1,000 hypotheses tested. A naive analysis suggests that each additional copy of a European ancestral allele at either the Xq13.1 or the Xq25 peak is independently associated with a BMI decrease of ∼0.1 Z-score units on average (equivalent to ∼0.64 kg/m^2^ and accounting for 0.3% of the variance in BMI, after adjusting for age, age-squared, sex and study). The true genetic effects are expected to be somewhat weaker because of discovery bias.

**Table 3:** Linear regression analysis of BMI on local European ancestry at the three admixture peaks.

	Model 1: Local ancestry only			Model 2: Local ancestry, ancestry from autosomes as a covariate			Model 3: Local ancestry, ancestry from the X chromosome as a covariate		
Admixture peaks	Reg. Coef.	(95% CI)	*P* value	Reg. Coef.	(95% CI)	*P* value	Reg. Coef.	(95% CI)	*P* value
5q13.3	0.03	(−0.01, 0.06)	0.071	0.09	(0.06, 0.13)	5.8×10^−7^	-	-	-
Xq13.1									
Both sexes	−0.13	(−0.16, −0.09)	2.2×10^−11^	−0.11	(−0.14, 0.07)	1.9×10^−7^	−0.10	(−0.14, 0.06)	2.2×10^−6^
Males	−0.15	(−0.23, −0.08)	3.5×10^−5^	−0.16	(−0.23, −0.08)	4.0×10^−5^	−0.16	(−0.24, −0.08)	6.4×10^−5^
Females	−0.10	(−0.14, −0.06)	7.2×10^−6^	−0.06	(−0.10, −0.01)	0.022	−0.04	(−0.09, 0.01)	0.089
	*P* for heterogeneity between sexes^a^ = 0.676			*P* for heterogeneity between sexes^a^ = 0.026			*P* for heterogeneity between sexes^a^ = 0.016		
Xq25									
Both Sexes	−0.13	(−0.17, −0.09)	4.5×10^−10^	−0.10	(−0.15, −0.06)	4.1×10^−6^	−0.10	(−0.14, −0.05)	4.3×10^−5^
Males	−0.11	(−0.19, −0.03)	0.008	−0.11	(−0.20, −0.03)	0.011	−0.11	(−0.19, −0.02)	0.017
Females	−0.12	(−0.16, −0.07)	5.2×10^−6^	−0.06	(−0.12, −0.01)	0.022	−0.05	(−0.11, 0.01)	0.091
	*P* for heterogeneity between sexes^a^ = 0.882			*P* for heterogeneity between sexes^a^ = 0.358			*P* for heterogeneity between sexes^a^ = 0.281		

Reg. Coef., regression coefficient: the change in Z score for each additional copy of the European ancestry allele; CI, confidence interval. In both-sex-combined analysis, BMI was adjusted for age, age-squared, sex and study, and then normal-quantile transformed. Sex-stratified analysis was performed in each gender group and thus not adjusted for sex.

a By Z test for difference between the two the regression coefficients.

The association at the 5q13.3 peak was particularly interesting in that it did not achieve statistical significance until the genome-wide estimate of European ancestry was added into the analysis. This was presumably because the locus effect was in the opposite direction to the genome-wide ancestry effect (thus, the effects cancel in the unadjusted analysis). Each additional copy of a European ancestral allele at 5q13.3 was significantly (*P* = 5.8×10^−7^) associated with an increase in BMI of 0.09 Z-score units (naively equal to ∼0.59 kg/m^2^, accounting for 0.3% of the variance in BMI), which was nominally significant (*P* = 5.8×10^−4^) after correcting for the approximately 1,000 independent hypotheses tested.

For the two peaks on chromosome X, we further examined whether the effects of the local ancestry on BMI were modified by gender. The local ancestry at Xq13.1 tended to be more strongly associated with BMI in males than in females. After adjusting for genome-wide European ancestry, the gender difference at Xq13.1 was significant (*P* for heterogeneity = 0.026, Model 2 in Table 3), which was in line with our results of dichotomous admixture scans. At Xq25, the effects of local ancestry did not show significant heterogeneity (*P*>0.05) between the two gender groups, either before or after adjusting for genome-wide European ancestry. A potential mechanism for the difference in the strength of association in men and women at the Xq13.1 locus is that women carry two copies of chromosome X whereas men carry only one, and hence this may simply reflect a difference in the genetics of the two genders on chromosome X.

Since our analysis pooled data from 14 studies, we also examined whether the strength of the admixture associations to BMI on chromosomes X and 5 differed across studies. Local ancestry estimates at each of the three admixture peaks were used to check for homogeneity of their correlation with BMI across studies. There was no evidence of heterogeneity across studies (all *P*>0.05, *I^2^* = 0%) at any of the three peaks (Table S2).

### 95% Credible Interval for the Three Loci

We constructed 95% CI for each of the three loci identified. The 95% CI for the chromosome 5 locus spanned from 69.2 to 77.2 Mb (an ∼8 Mb region) on build 35 of the human genome reference sequence. The 95% CI for the higher admixture peak on chromosome X spanned from 114.4 to 124.4 Mb (an ∼10 Mb region), and then 95% CI for the other chromosome X admixture peak spanned from 47.8 to 89.2 Mb, a much broader region (∼40 Mb).

## Discussion

We have carried out admixture mapping analyses to search for genomic regions associated with BMI. This pooled analysis of samples from 14 studies is the largest admixture scan reported to date. In more than 15,000 individuals, we identified a locus on chromosome 5 where greater local European ancestry was associated with higher levels of BMI (*P* = 5.8×10^−7^), and two regions on chromosome X where greater local European ancestry was associated with lower levels of BMI (both *P*<5.0×10^−6^). Each of these three associations was above and beyond the contribution of genome-wide European ancestry, and each reached genome-wide significance.

One of the major strengths of this study is its large sample size, with over 15,000 African Americans. However, the large sample also introduced complications in that it required the pooling of several studies which potentially introduced various types of heterogeneity to the study samples. For example, we included individuals with either self-reported BMI or clinically measured BMI in the present study. It is well known that individuals tend to under report their body weight and that this measurement error is potentially more common among heavier individuals. Moreover, this type of measurement error can reduce the statistical power of a study. To assess the potential effects of such measurement error, we performed subgroup analysis by restricting the samples to those from the six population-based cohort studies, where body weight and height were clinically measured according to study protocols (samples in the six cohorts represented 94% of all samples with measured BMI) and found the two sets of results to be largely comparable. Additional subgroup analyses, as shown in Table 2, also confirmed the robustness of our findings [21],[25],[26].

The inverse correlation between BMI and percentage of European ancestry estimated on the genome-wide scale confirmed the results from previous studies of smaller sample size and fewer markers [29],[30]. However, while genome-wide ancestry is likely correlated with local ancestry, it cannot fully capture ancestry information at each locus as there exists variation across the genome in the effects of locus-specific ancestry on obesity. In particular, local European ancestry at 5q13.3 was positively associated with BMI, providing the first evidence of a genome-wide significant ancestry association being in the opposite direction to the overall epidemiological association.

The 95% CI for the chromosome 5 peak harbors a number of genes, including the cocaine and amphetamine regulated transcript (*CART*) gene, which is a candidate for modulating obesity. CART is a hypothalamic neuropeptide that transmits a physiological anorexigenic signal and is involved in appetite regulation [31],[32]. Experiments have also shown that *CART* knock-out mice have increased body weight compared with wild type mice [33]. Genomic regions containing the *CART* gene have also been linked to both BMI and serum leptin levels in a study of French Caucasian families [34]. SNPs in the 5′ upstream region have been reported to be associated with obesity in Japanese [35] and French [36]. However, association studies in European-related populations [37],[38] and Pima Indians [39] have not found associations between BMI and the *CART* gene in these populations, and to our knowledge no published studies have studied *CART* variants in African Americans. Further mapping work is needed to determine whether the *CART* gene or other genetic variants in the interval may influence the risk of obesity.

There have been very few studies reporting linkage of obesity with markers on the X chromosome [40], yet three prior studies also reported either suggestive or significant linkage of obesity to the q arm of chromosome X [41]–[43]. Although these three studies were performed in European-American families, they all mapped the obesity locus to the Xq23–q24 region, which overlaps with the 95% CI of the highest admixture peak on chromosome X in our study. The 95% CI in our study contains one particular gene that may be a candidate for obesity susceptibility. The gene *s*olute carrier family 6 member 14 (*SLC6A14*) is involved in serotonin synthesis and serotonergic receptor mechanisms that have been implicated in appetite control and body weight regulation [44]–[46]. Nominally significant evidence of association between BMI and a SNP (22510C/G) in *SLC6A14* was observed in ∼1,800 samples from Finland and Sweden (*P* = 0.003), and females were found to contribute most to this particular observed association [43]. The gender difference observed in the previous study [43] is in line with the results from our dichotomous admixture scans at this locus, although the difference observed between men and women in our study did not reach statistical significance. Another potential candidate gene near the highest admixture peak is the cullin 4B (*CUL4B*) gene. *CUL4B* was recently identified as a causative gene for an X-linked mental retardation syndrome, which was associated with several clinical features, including central obesity [47].

Although we did not detect a significant association in the region of the *FTO* gene, we noticed that the second highest admixture peak (locus-specific LOD = 3.68) identified in our quantitative scans was on chromosome 16, about 5.6 Mb away from the *FTO* gene, and its 99% CI spanned 51.5 to 66.8 Mb (on build 35 of the human reference sequence), which is a region that includes the *FTO* gene. (However, *FTO* is outside the 95% CI.) Further fine-mapping analysis may determine whether additional variations in *FTO* may explain the intriguing admixture signal in this region. The melanocortin-4 receptor (*MC4R*) gene, located on chromosome 18q21.32, is the second obesity-susceptibility gene discovered by genome-wide association studies in individuals of European origin [48],[49]. However, our dichotomous and quantitative admixture scans did not identify any admixture signals on chromosome 18q.

In summary, we have carried out a genome-wide admixture mapping scan in 15,280 African Americans and have identified three loci, 5q13.3, Xq13.1 and Xq25, that may harbor genetic variants associated with variations in BMI. The local ancestry associations to BMI at each of the three admixture-generated peaks were statistically significant, suggesting the presence of a genetic effect at these loci above and beyond the effects of genome-wide ancestry. Follow-up fine mapping and focused analysis of each locus using data that emerge from genome-wide association studies in African Americans with measured BMI will be crucial to determine whether these regions harbor genetic variants predisposing to obesity.

The present study is also methodologically significant in illustrating how searches for genes in African Americans and diverse populations can result in the detection of genetic loci that have eluded discovery in European-derived populations, perhaps because the underlying variants are too rare in the latter populations. However, there is no reason to think that the three loci we have identified are biologically important only in African Americans. Replication and fine-mapping studies in other ethnic groups, including Hispanic Americans and Pacific Islanders, with a similar risk of obesity to African Americans, and even European Americans and East Asians with a lower but still important rate of this condition, may further elucidate these regions of the genome. Studying multiple populations to fine-map a locus highlighted in an admixture scan can be more informative than studying any one population, as was previously demonstrated by our use of a multi-ethnic cohort to fine-map prostate cancer risk factors at 8q24 [50].

## Supporting Information

Table S1 Interesting chromosome regions, other than Xq25, Xq13.1 and 5q13.3, observed in the dichotomous admixture scans for BMI.
(0.01 MB PDF)

Table S2 Correlations between local European ancestry and BMI by study.
(0.14 MB PDF)

Text S1 Description of each study.
(0.04 MB PDF)

Text S2 Details of quantitative trait analysis.
(0.07 MB PDF)
