# Supplementary material for: Admixture Mapping of 15,280 African Americans Identifies Obesity Susceptibility Loci on Chromosomes 5 and X
Source: PLoS Genet. 2009 May 22;5(5):e1000490. doi: 10.1371/journal.pgen.1000490 (PMC2679192; doi:10.1371/journal.pgen.1000490)
Supplement: Table S1 — Interesting chromosome regions, other than Xq25, Xq13.1 and 5q13.3, observed in the dichotomous admixture scans for BMI. (0.01 MB PDF) [file pgen.1000490.s001.pdf]

Table S1. Interesting chromosome regions, other than Xq25, Xq13.1 and 5q13.3, observed in the dichotomous admixture scans for BMI

Locus	Locus-specific LOD	Case-control Z score
2p22.2	-0.17	-3.78[a]
2p16.1	2.65[b]	-3.13[a]
4q28.3	0.76	-3.60[a]
4q34.1	1.04	-3.63[a]
7q11.21	-1.89	-3.46[a]
8p11.22	-0.25	3.34[a]
14q13.3	-2.38	3.08[a]
16q21	-1.38	3.68[a]

[a] Loci determined by a case-control Z score.
[b] Loci determined by a locus-specific LOD.
