# Supplementary material for: Admixture Mapping of 15,280 African Americans Identifies Obesity Susceptibility Loci on Chromosomes 5 and X
Source: PLoS Genet. 2009 May 22;5(5):e1000490. doi: 10.1371/journal.pgen.1000490 (PMC2679192; doi:10.1371/journal.pgen.1000490)
Supplement: Table S2 — Correlations between local European ancestry and BMI by study. (0.14 MB PDF) [file pgen.1000490.s002.pdf]

Table S2. Correlations between local European ancestry and BMI by study

		Local ancestry at 5q13.3		Local ancestry at Xq13.1		Local ancestry at Xq25	
**Study**	**n**	**ρ**	**SE**	**ρ**	**SE**	**ρ**	**SE**
ARIC	3,522	-0.004	0.017	-0.070	0.018	-0.061	0.017
BCFR	268	-0.003	0.060	-0.020	0.053	-0.009	0.065
CARE	365	-0.003	0.055	-0.085	0.052	-0.079	0.052
DHS	1,718	0.048	0.025	-0.028	0.024	-0.046	0.023
FIND	1,445	0.019	0.027	-0.044	0.028	-0.047	0.028
GCI	503	0.044	0.041	-0.048	0.048	-0.029	0.045
Health ABC	1,172	-0.006	0.029	-0.069	0.028	-0.038	0.027
JHS	2,141	0.008	0.022	-0.060	0.020	-0.070	0.020
LIFE	108	-0.144	0.110	-0.022	0.087	-0.069	0.105
MEC	3,199	0.016	0.018	-0.067	0.018	-0.046	0.018
MrOS	199	-0.002	0.070	-0.016	0.070	0.005	0.069
SFBABCS	152	0.108	0.076	-0.063	0.082	-0.088	0.084
SOF	368	-0.023	0.052	-0.070	0.046	-0.099	0.050
WCHS	120	-0.116	0.117	-0.131	0.096	-0.191	0.081
Total	15,280	0.014	0.008	-0.056	0.008	-0.052	0.008
**Tests of heterogeneity**		$\chi^2 = 9.68$	$P = 0.720$	$\chi^2 = 4.65$	$P = 0.982$	$\chi^2 = 7.23$	$P = 0.890$
			$I^2 = 0\%$		$I^2 = 0\%$		$I^2 = 0\%$

ρ, correlation coefficient; SE, standard error; I^2, inconsistency metric. BMI was adjusted for age, age-squared and sex, and then normal-quantile transformed in each study.

ARIC, Atherosclerosis Risk in Communities Study; BCFR, Breast Cancer Family Registry; CARE, Los Angeles component of the Women's Contraceptive and Reproductive Experiences Study, DHS, Dallas Heart Study; FIND, Family Investigation of Nephropathy and Diabetes Study; GCI, Genomics Collaborative Study; Health ABC, Health, Aging and Body Composition Study; JHS, Jackson Heart Study; LIFE, Learning the Influence of Family and the Environment Study; MEC, Multiethnic Cohort of Los Angeles and Hawaii; MrOS, Osteoporotic Fractures in Men Study; SFBABCS, the San Francisco Bay Area Breast Cancer Study; SOF, Study of Osteoporotic Fractures; WCHS, Women's Circle of Health Study.
