# Supplementary material for: Admixture Mapping of 15,280 African Americans Identifies Obesity Susceptibility Loci on Chromosomes 5 and X
Source: PLoS Genet. 2009 May 22;5(5):e1000490. doi: 10.1371/journal.pgen.1000490 (PMC2679192; doi:10.1371/journal.pgen.1000490)
Supplement: Text S1 — Description of each study. (0.04 MB PDF) [file pgen.1000490.s003.pdf]

Admixture mapping of 15,280 African Americans identifies obesity susceptibility loci on chromosomes 5 and X

Supplementary Methods

Text S1. Description of each study

ARIC. A total of 3,522 samples were derived from the Atherosclerotic Risk in Communities Study, which is a community-based prospective study that searches for risk factors of clinical and subclinical atherosclerotic disease in a cohort of 15,792 persons, including 4,266 African Americans [1]. Approximately 88% of the ARIC African Americans came from the study center in Jackson, MS and 12% from Forsyth County, NC. The genotyping for this study was carried out using genomic DNA on the Phase 3 marker panel at the Center for Inherited Disease Research in Baltimore. Of the African Americans, 3,522 had both high-quality genotype data and a BMI measurement.

BCFR. A total of 268 samples were derived from the Northern California site of the Breast Cancer Family Registry, an international resource for genetic studies of breast cancer [2]. Incident cases of invasive breast cancer aged 18-64 years diagnosed between 1995 and 2003 were ascertained from the population-based cancer registry of the Greater San Francisco Bay area. The present analysis included cases who self-identified as African American. The genotyping for this study was carried out at the University of California, San Francisco using genomic DNA on the Phase 2 marker panel.

CARE. A total of 365 African-American samples were derived from the Los Angeles component of the Women's Contraceptive and Reproductive Experiences Study, a population-based case-control study of incident first primary invasive breast cancer diagnosed between 1994 and 1998 among African-American and Caucasian women aged 35 to 64 years [3]. The present analysis included cases who were identified by the Cancer Surveillance Program for Los Angeles County, a member of California's statewide Cancer Registry and one of the National Cancer Institutes' Surveillance, Epidemiology and End Results (SEER) registries. The genotyping for this study was carried out using whole genome amplified DNA on the Phase 3 marker panel at the University of Southern California.

DHS. A total of 1,718 samples were derived from the DHS, which was designed as a multiethnic, population-based sample based in Dallas County, Texas to investigate the underlying mechanisms contributing to ethnic differences in cardiovascular health at the community level [4]. The genotyping for this study was carried out using genomic DNA on the Phase 1 marker panel at the University of Texas, Southwestern.

FIND. A total of 1,445 samples were derived from the Johns Hopkins site of the Family Investigation of Nephropathy and Diabetes Study, which is a multicenter study designed to identify genetic determinants of kidney disease in four ethnic groups [5]. For the admixture mapping studies, African-American patients with end-stage renal disease and their spouses/partners unaffected by nephropathy were recruited, and for this admixture analysis, we only included participants recruited from Johns Hopkins University in Baltimore. The genotyping for this study was carried out using genomic DNA on the Phase 3 marker panel at the Laboratory of Genomic Diversity at the National Cancer Institute.

GCI. A total of 503 samples were derived from a population-based collection carried out by Genomics Collaborative Inc. at multiple sites across the U.S., sampling individuals with clinically significant primary hypertension. The genotyping for this study was done using genomic DNA on the Phase 2 marker panel at the Broad Institute of Harvard and MIT.

Health ABC. A total of 1,172 African-American samples were derived from the Health, Aging and Body Composition Study, which consists of 3,075 men and women aged 70-79 who were enrolled between April 1997 and June 1998; of them 42% are African American [6]. All were Medicare beneficiaries living near Pittsburgh or Memphis and all reported no difficulty in performing basic physical activities. The genotyping for this study was done using whole genome amplified DNA [7] on the Phase 2 marker panel at the Broad Institute of Harvard and MIT.

JHS. A total of 2,141 samples were derived from the Jackson Heart Study, a long-term, community-based observational study of cardiovascular disease and its risk factors in African Americans residing in the tri-county area around Jackson, Mississippi [8]. Approximately 30% of JHS participants had previously participated in ARIC. Thus, we excluded JHS participants who had been included in our ARIC sample, as well as those with incomplete phenotypic or genetic data. Because JHS includes a nested family sample, we randomly dropped individuals from families until we retained only one individual per family for analysis. The genotyping for this study was done using genomic DNA on either the Phase 2 (n = 524 samples) or Phase 3 (n = 1,617 samples) marker panels, at the Broad Institute of Harvard and MIT.

LIFE. A total of 108 samples were derived from the Learning the Influence of Family and the Environment Study, which included invasive African-American breast cancer cases from Los Angeles County aged 20 to 49 years [9]. Incident cases diagnosed between 2000 and 2003 were identified from the Los Angeles SEER registry. The genotyping for this study was carried out using whole genome amplified DNA on the Phase 3 marker panel at the University of Southern California.

MEC. A total of 3,199 samples were derived from the Multiethnic Cohort of Los Angeles and Hawaii, a prospective cohort that includes over 215,000 individuals of whom 16.3% are African American. The MEC samples in our analysis were not a random cross-section of the population; instead, they included an oversampling of individuals with prostate cancer, type 2 diabetes, breast cancer and hypertension, whom we had admixture scanned for other studies focusing on these phenotypes [10,11]. The genotyping for this study was done using genomic DNA on the Phase 1 (n = 1,300 samples), Phase 2 (n = 1,476 samples) and Phase 3 (n = 423 samples) marker panels at the Broad Institute of Harvard and MIT and the University of Southern California.

MrOS. A total of 199 samples were derived from the Osteoporotic Fractures in Men Study, a prospective cohort study designed to determine risk factors for osteoporosis and fractures in men at least 65 years of age [12]. Men were recruited from population-based listings between 2000 and 2002 in six areas of the U.S.: Birmingham, AL; the Monongahela Valley near Pittsburgh, PA; Minneapolis, MN; Palo Alto, CA; San Diego, CA; and Portland, OR [13]. The genotyping for this study was done using whole genome amplified DNA from dried blood spots on the Phase 3 marker panel at the Broad Institute of Harvard and MIT.

SFBABCS. A total of 152 samples were derived from the San Francisco Bay Area Breast Cancer Study, a multiethnic population-based case-control study of breast cancer [14]. Incident cases of invasive breast cancer aged 35-79 years diagnosed between 1995 and 2002 were ascertained from the population-based cancer registry of the Greater San Francisco Bay Area. The present analysis included cases diagnosed between 1997 and 1999 who self-identified as African American. The genotyping for this study was carried out at the University of California, San Francisco using genomic DNA on the Phase 2 marker panel.

SOF. A total of 368 samples were derived from the Study of Osteoporotic Fractures, a prospective cohort study carried out on community-dwelling elderly women aged 65 and older to assess their risk for osteoporosis [15]. Participants were recruited from four areas in the U.S.: Baltimore, MD; Pittsburgh, PA; Minneapolis, MN and Portland, OR. In 1997, a cohort of elderly African-American women with functional characteristics that were similar to the baseline SOF sample was added. The genotyping for this study was done using whole genome amplified DNA from dried blood spots on the Phase 3 marker panel at the Broad Institute of Harvard and MIT.

WCHS. A total of 120 samples were derived from the Women's Circle of Health Study, an ongoing case-control study to determine predictors of early, aggressive breast cancer in African-American and Caucasian women. Cases 20 to 65 years of age were identified from major hospitals in the New York City metropolitan region, and, in collaboration with the New Jersey State Cancer Registry, through rapid case ascertainment at all major hospitals in seven counties in New Jersey. For the present analysis, only women with breast cancer who self-reported as African American were included. The genotyping for this study was carried out using genomic

DNA obtained from whole blood or saliva on the Phase 2 marker panel at the University of California, San Francisco.

References

1. The ARIC Investigators (1989) The Atherosclerosis Risk in Communities (ARIC) Study: design and objectives. The ARIC investigators. Am J Epidemiol 129: 687-702.

2. John EM, Hopper JL, Beck JC, Knight JA, Neuhausen SL et al. (2004) The Breast Cancer Family Registry: an infrastructure for cooperative multinational, interdisciplinary and translational studies of the genetic epidemiology of breast cancer. Breast Cancer Res 6: R375-R389.

3. Marchbanks PA, Mcdonald JA, Wilson HG, Burnett NM, Daling JR et al. (2002) The NICHD Women's Contraceptive and Reproductive Experiences Study: methods and operational results. Ann Epidemiol 12: 213-221.

4. Victor RG, Haley RW, Willett DL, Peshock RM, Vaeth PC et al. (2004) The Dallas Heart Study: a population-based probability sample for the multidisciplinary study of ethnic differences in cardiovascular health. Am J Cardiol 93: 1473-1480.

5. Knowler WC, Coresh J, Elston RC, Freedman BI, Iyengar SK et al. (2005) The Family Investigation of Nephropathy and Diabetes (FIND): design and methods. J Diabetes Complications 19: 1-9.

6. Visser M, Newman AB, Nevitt MC, Kritchevsky SB, Stamm EB et al. (2000) Reexamining the sarcopenia hypothesis. Muscle mass versus muscle strength. Health, Aging, and Body Composition Study Research Group. Ann N Y Acad Sci 904: 456-461.

7. Hosono S, Faruqi AF, Dean FB, Du Y, Sun Z et al. (2003) Unbiased whole-genome amplification directly from clinical samples. Genome Res 13: 954-964.

8. Taylor HA, Jr. (2003) Establishing a foundation for cardiovascular disease research in an African-American community--the Jackson Heart Study. Ethn Dis 13: 411-413.

9. Ma H, Bernstein L, Ross RK, Ursin G (2006) Hormone-related risk factors for breast cancer in women under age 50 years by estrogen and progesterone receptor status: results from a case-control and a case-case comparison. Breast Cancer Res 8: R39.

10. Freedman ML, Haiman CA, Patterson N, McDonald GJ, Tandon A et al. (2006) Admixture mapping identifies 8q24 as a prostate cancer risk locus in African-American men. Proc Natl Acad Sci U S A 103: 14068-14073.

11. Deo RC, Patterson N, Tandon A, McDonald GJ, Haiman CA et al. (2007) A High-Density Admixture Scan in 1,670 African Americans with Hypertension. PLoS Genet 3: e196.

12. Orwoll E, Blank JB, Barrett-Connor E, Cauley J, Cummings S et al. (2005) Design and baseline characteristics of the osteoporotic fractures in men (MrOS) study--a large observational study of the determinants of fracture in older men. Contemp Clin Trials 26: 569-585.

13. Blank JB, Cawthon PM, Carrion-Petersen ML, Harper L, Johnson JP et al. (2005) Overview of recruitment for the osteoporotic fractures in men study (MrOS). Contemp Clin Trials 26: 557-568.

14. John EM, Schwartz GG, Koo J, Wang W, Ingles SA (2007) Sun exposure, vitamin D receptor gene polymorphisms, and breast cancer risk in a multiethnic population. Am J Epidemiol 166: 1409-1419.

15. Cummings SR, Black DM, Nevitt MC, Browner WS, Cauley JA et al. (1990) Appendicular bone density and age predict hip fracture in women. The Study of Osteoporotic Fractures Research Group. JAMA 263: 665-668.
