# Supplementary material for: Admixture Mapping of 15,280 African Americans Identifies Obesity Susceptibility Loci on Chromosomes 5 and X
Source: PLoS Genet. 2009 May 22;5(5):e1000490. doi: 10.1371/journal.pgen.1000490 (PMC2679192; doi:10.1371/journal.pgen.1000490)
Supplement: Text S2 — Details of quantitative trait analysis. (0.07 MB PDF) [file pgen.1000490.s004.pdf]

Admixture mapping of 15,280 African Americans identifies obesity susceptibility loci on chromosomes 5 and X

Supplementary Methods

Text S2. Details of quantitative trait analysis

A major challenge in admixture mapping is to be able to scan not only for a trait that is dichotomous (such as healthy or having a disease), but also to identify associations of ancestry to a quantitative trait (QTL) such as systolic or diastolic blood pressure, an individual's HDL or triglyceride level, or BMI. The ANCESTRYMAP software was originally designed to analyze a dichotomous trait, but here, we extend it to allow mapping of a QTL.

For our admixture mapping analysis we use a full Bayesian model, and hence we need to explicitly model how ancestry confers risk for disease. For our model of quantitative trait risk we assume as we did for a dichotomous trait in Patterson et al., 2004 [1] that $\theta(i)$ is the proportion of European ancestors for individual i, and a is the proportion at a locus of interest (this can be either 0, 1 or 2 European alleles). We assume as for the dichotomous trait analysis that in our population of African Americans, θ is Beta distributed. Our past experience shows that this is a reasonable, albeit crude, approximation. We then explore how well an individual's value for a quantitative trait $q(i)$ is predicted by their local ancestry state a, controlling for their overall proportion of ancestry $\theta(i)$. We make the assumption that the quantitative trait $q(i)$ for individual i is normally distributed, which can be enforced for a QTL by applying a non-parametric 'probit'

transformation; that is, rank-ordering all observations and then applying an inverse normal transformation.

Our strategy for searching for a QTL locus is to compute a Bayes Factor F at each MCMC iteration and at each locus in the genome, which scores for the locus being associated to the trait, versus not associated. We then average F across iterations, as we do with a dichotomous trait, to get an overall assessment of the weight of evidence for association at the locus.

To be more explicit about our model, we set for individual i at a particular locus:

$$q(i) = c_0 n_0 + c_1(\theta(i) - \mu) + c_2(a - 2\theta(i)) \tag{1}$$

Here, μ is the mean of θ for the entire population, a is the number of European chromosomes at the locus, which has mean $2\theta(i)$, and $c_0 n_0$ is a Gaussian residual term. We assume n_0 is standard normal, so that the standard deviation of the residual is c_0. We are interested in detecting association with ancestry at a particular locus in the genome, and not just with the average ancestry across the genome. Our Bayesian scoring carefully deals with this by building the average genome ancestry component into the null model. Thus, in equation (1), the second term deals with the overall effect on the QTL of an individual's ancestry being different from the population average, and the third term deals with the effect of the local ancestry state compared to its expectation $2\theta(i)$, in the absence of an association to the QTL.

We will assume that the correlation between genome-wide ancestry θ and the quantitative trait q is known. As part of our Bayesian model, we then specify the fraction of the remaining quantitative trait variance explained by the locus. This is similar to specifying the risk factor

associated with a disease locus as we do in our original discrete trait analysis [1]. Easy formulae from the theory of the Beta distribution now allow us to calculate c_0, c_1, and c_2.

To calculate the likelihood, we note that the probability density of $q(i)$ is given as:

$$P(q) = \sum_a Prob(a) N\left(q; c_1(\theta - \mu) + c_2(a - \theta), c_0^2\right) \qquad (2)$$

where $N(x; m, v)$ is the density of a normal variable, mean m variance v. We will take the *null model* to be the above model with $c_2 = 0$. Thus under the null, local ancestry does not affect the observation q. The MCMC that we will implement samples model parameters from the posterior distribution under the null. This is only slightly more complex than the MCMC of Patterson et al.[1] The Bayesian scoring can then be carried out just as in the dichotomous trait analysis, but using equation (2). We omit the easy details.

Summarizing, equation (1) is a direct 'generative' model for the quantitative trait. Natural extensions of the Bayesian methods in ANCESTRYMAP for dichotomous traits are then easy to derive and implement.

References

1. Patterson N, Hattangadi N, Lane B, Lohmueller KE, Hafler DA et al. (2004) Methods for high-density admixture mapping of disease genes. Am J Hum Genet 74: 979-1000.
